# The projected health and economic impact of increased colorectal cancer screening participation among Canadians by income quintile

**DOI:** 10.17269/s41997-024-00868-8

**Published:** 2024-03-19

**Authors:** Abisola A. Adegbulugbe, Eliya Farah, Yibing Ruan, Jean H. E. Yong, Winson Y. Cheung, Darren R. Brenner

**Affiliations:** 1 Department of Oncology, Cumming School of Medicine, University of Calgary, Calgary, AB Canada; 2 Department of Community Health Sciences, University of Calgary, Calgary, AB Canada; 3 Department of Cancer Epidemiology and Prevention Research, Alberta Health Services, Calgary, AB Canada; 4 Canadian Partnership Against Cancer, Toronto, ON Canada; 5 Forzani & MacPhail Colon Cancer Screening Centre, Alberta Health Services, Calgary, AB Canada

**Keywords:** Canada, Colorectal neoplasms, Early detection of cancer, Income, Mass screening, Socioeconomic factors, Canada, tumeurs colorectales, dépistage précoce du cancer, revenu, dépistage de masse, facteurs socioéconomiques

## Abstract

**Objectives:**

Disparities in colorectal cancer (CRC) screening uptake by socioeconomic status have been observed in Canada. We used the OncoSim-Colorectal model to evaluate the health and economic outcomes associated with increasing the participation rates of CRC screening programs to 60% among Canadians in different income quintiles.

**Methods:**

Baseline CRC screening participation rates were obtained from the 2017 Canadian Community Health Survey. The survey participants were categorized into income quintiles using their reported household income and 2016 Canadian Census income quintile thresholds. Within each quintile, the participation rate was the proportion of respondents aged 50–74 who reported having had a fecal test in the past two years. Using the OncoSim-Colorectal model, we simulated an increase in CRC screening uptake to 60% across income quintiles to assess the effects on CRC incidence, mortality, and associated economic costs from 2024 to 2073.

**Results:**

Increasing CRC screening participation rates to 60% across all income quintiles would prevent 69,100 CRC cases and 36,600 CRC deaths over 50 years. The improvement of clinical outcomes would also translate to increased person-years and health-adjusted person-years. The largest impact was observed in the lowest income group, with 22,200 cases and 11,700 deaths prevented over 50 years. Increased participation could lead to higher screening costs ($121 million CAD more per year) and lower treatments costs ($95 million CAD less per year), averaged over the period 2024–2073.

**Conclusion:**

Increased screening participation will improve clinical outcomes across all income groups while alleviating associated treatment costs. The benefits of increased participation will be strongest among the lowest income quintile.

**Supplementary Information:**

The online version contains supplementary material available at 10.17269/s41997-024-00868-8.

## Résumé

**Objectifs:**

Des disparités dans le recours au dépistage du cancer colorectal (CCR) selon le statut socioéconomique sont observées au Canada. Nous avons utilisé le modèle OncoSim-Colorectal pour évaluer les résultats cliniques et économiques associés à une augmentation à 60 % des taux de participation aux programmes de dépistage du CCR chez les Canadiennes et les Canadiens appartenant à différents quintiles de revenu.

**Méthode:**

Les taux de participation de référence au dépistage du CCR provenaient de l’Enquête sur la santé dans les collectivités canadiennes de 2017. Nous avons catégorisé les participantes et les participants de l’enquête en quintiles de revenu à l’aide du revenu du ménage déclaré et des seuils de quintiles de revenu du Recensement du Canada de 2016. Dans chaque quintile, le taux de participation était la proportion des répondantes et des répondants de 50 à 74 ans ayant dit avoir subi un test fécal au cours des deux années antérieures. À l’aide du modèle OncoSim-Colorectal, nous avons simulé une augmentation à 60 % du recours au dépistage du CCR dans tous les quintiles de revenu pour en évaluer les effets sur l’incidence, la mortalité et les coûts économiques associés du CCR entre 2024 et 2073.

**Résultats:**

L’augmentation des taux de participation au dépistage du CCR à 60 % dans tous les quintiles de revenu préviendrait 69 100 cas de CCR et 36 600 décès dus au CCR sur 50 ans. L’amélioration des résultats cliniques se traduirait aussi par une augmentation des personnes-années et des personnes-années corrigées en fonction de la santé. Nous avons observé l’effet le plus marquant dans la catégorie de revenu inférieure, avec la prévention de 22 200 cas et de 11 700 décès sur 50 ans. La participation accrue pourrait entraîner une hausse des coûts de dépistage (121 millions de dollars canadiens de plus par année) et une baisse des coûts de traitement (95 millions de dollars canadiens de moins par année), en moyenne, sur la période de 2024 à 2073.

**Conclusion:**

La participation accrue au dépistage améliorera les résultats cliniques dans toutes les catégories de revenu tout en réduisant les coûts de traitement associés. Les avantages d’une participation accrue seront les plus marquants dans le quintile de revenu inférieur.

## Introduction

Colorectal cancer (CRC) is the fourth most commonly diagnosed cancer and the second leading cause of cancer-related deaths in Canada (Brenner et al., 2022; Ruan et al., 2023). In 2022, it is estimated that there were 24,300 new CRC diagnoses and 9400 CRC-related deaths in Canada (Brenner et al., 2022). Across Canada, the adoption of CRC screening programs has contributed to the steady decline in CRC incidence rates (Demers et al., 2022). Currently, CRC screening programs have been implemented in most of Canada, with the earliest province-wide program launched in 2008 (Canadian Partnership Against Cancer, 2022; Schreuders et al., 2015). The CRC screening guidelines, published by the Canadian Task Force on Preventive Health Care (CTFPHC) in 2016, recommend that average-risk asymptomatic individuals between the ages of 50 and 74 be screened with a fecal occult blood test (guaiac fecal occult blood test or fecal immunochemical test) every 24 months or flexible sigmoidoscopy every 10 years (Canadian Task Force on Preventive Health, 2016).

The declines in CRC incidence and mortality rates in Canada are largely attributable to the early detection and removal of precancerous polyps through organized CRC screening programs (Komanduri et al., 2022). While these programs have reduced the burden of CRC in Canada, disparities in CRC screening uptake continue to persist. In particular, income has been strongly correlated with screening uptake, with lower participation among individuals of a lower household income (Kiran et al., 2017). Several studies have demonstrated the association between lower income and having never been screened with either a stool test or a colonoscopy/sigmoidoscopy (Blair et al., 2019; Decker & Singh, 2014). In addition, prior analyses of 2007–2016 data from Canadian Community Health Survey (CCHS) datasets have indicated that individuals who have not undergone any CRC screening tests or procedures were more likely to have an income below $20,000 CAD (Abdel-Rahman, 2021). Therefore, policies aimed at increasing CRC screening uptake would likely be most beneficial for Canadians with low income. However, the health and economic outcomes associated with increasing the CRC screening participation rates, particularly across the different income groups, are not known.

In this study, we modeled and evaluated the impact of increasing CRC screening participation to the national target of 60% (Canadian Partnership Against Cancer, 2013) on important health and economic outcomes among Canadians with varying income levels.

## Methods

We used the OncoSim microsimulation platform (version 3.6.2.5), developed by Statistics Canada and the Canadian Partnership Against Cancer, to project the impact of increased CRC screening uptake among Canadians with varying levels of household income on CRC-related incidence, mortality, and the associated economic costs. The OncoSim model has been described in further detail in a previous publication by Gauvreau et al. (2017).

### OncoSim-Colorectal model

**Fig. 1:**
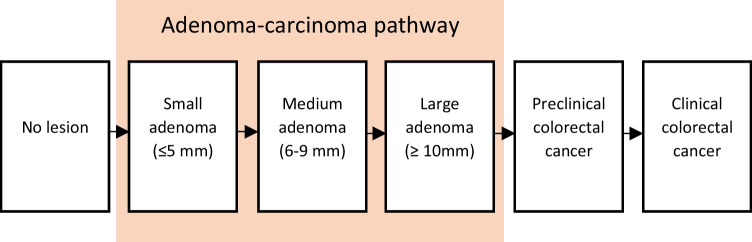
Natural history of colorectal cancer simulated by the OncoSim model. Adapted from: de Jonge, L., Worthington, J., van Wifferen, F., Iragorri, N., Peterse, E. F. P., Lew, J. B., Greuter, M. J. E., Smith, H. A., Feletto, E., Yong, J. H. E., Canfell, K., Coupé, V. M. H., Lansdorp-Vogelaar, I., & COVID-19 and Cancer Global Modelling Consortium working group 2 (2021). Impact of the COVID-19 pandemic on faecal immunochemical test-based colorectal cancer screening programmes in Australia, Canada, and the Netherlands: a comparative modelling study. *The Lancet. Gastroenterology & Hepatology, 6*(4), 304–314. https://doi.org/10.1016/S2468-1253(21)00003-0

A detailed description of the OncoSim-CRC model has been described previously (Coldman et al., 2015). Validation of the OncoSim-CRC model has been published in a previous publication by Coldman et al. (2015). Briefly, the OncoSim-CRC model simulates the natural history and progression of CRC based on the assumption that most CRC cases develop from adenomas via the adenoma-carcinoma pathway (Fig. 1); prevalence of adenomas was estimated from autopsy studies (de Jonge et al., 2021; Smith et al., 2021; Yong et al., 2021). Given that screening allows for the detection of cancers at an early, more treatable stage, the model assumes that the stage-specific cancer survival rates of CRCs detected through screening are better than those detected clinically (Shaukat et al., 2021; Yong et al., 2021). In our simulation, average-risk asymptomatic individuals between the ages of 50 and 74 were offered a fecal immunochemical test (FIT) for CRC screening every two years in Canada. We assumed 85% of those with positive FIT results (Supplemental File 1, Table 5) received a follow-up colonoscopy and that colonoscopies were 95% sensitive in detecting colorectal cancer.

### Screening participation and cancer incidence by income quintile

We obtained the baseline CRC screening participation rates by income quintile from the 2017 Canadian Community Health Survey (Statistics Canada, 2017). The survey participants were categorized into income quintiles according to their reported household income. The income quintile thresholds were obtained from the 2016 Canadian Census (Nkwinkeum et al., 2021). Within each quintile, the participation rate was estimated as the proportion who reported having had a fecal test in the past two years among all respondents aged 50–74. Because the cancer incidence rates differ across the income quintiles, we acquired age-standardized incidence rates (ASIR) of CRC from the Canadian Cancer Registry. We then calibrated the ASIR of CRC in the base case scenario of OncoSim by changing the “colorectal adenoma rates age multiplier” parameter, so that each income quintile had a base case scenario with their specific CRC incidence rate. Following calibration, we carried out simulations with the CRC screening participation rate either at status quo (baseline rate estimated from CCHS 2016) or 60% between 2024 and 2073 at a FIT threshold of 100 ng/ml. As a sensitivity analysis, we carried out simulations with a FIT threshold of 50 ng/ml or 175 ng/ml, which are used in some Canadian provinces. The positivity rate of FIT at 50, 100, or 175 ng/ml in detecting adenoma and cancer is shown in Supplemental File 1, Table 7.

### Outcomes

For this analysis, the following outcomes were evaluated: (1) incidence, defined as the number of new CRC diagnoses; (2) mortality, the number of CRC-related deaths; (3) economic costs, defined as the total costs in Canadian dollars associated with the screening program and cancer management; (4) incremental cost per HAPY, defined as the incremental cost associated with each health-adjusted person year gained from increasing the CRC screening participation rate to 60%.

## Results

### CRC cases and deaths

**Fig. 2:**
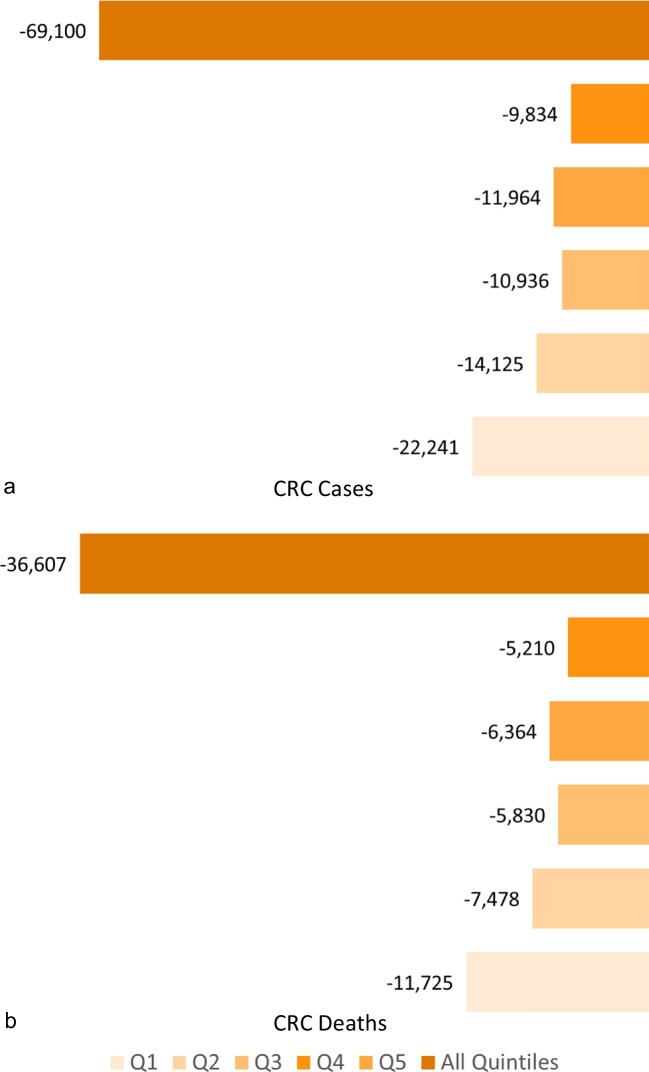
Difference between status quo and projected CRC incidence and mortality over a 50-year period (2024–2073) by income quintile in Canada. **a** Difference between status quo and projected CRC incidence over a 50-year period (2024–2073) by income quintile in Canada. **b** Difference between status quo and projected CRC mortality over a 50-year period (2024–2073) by income quintile in Canada. Q1 = Quintile 1, Q2 = Quintile 2, Q3 = Quintile 3, Q4= Quintile 4, Q5 = Quintile 5, All quintiles = Summation of quintiles 1–5

According to the CCHS 2017 survey, the CRC screening program participation rates from income quintile 1 (lowest) to 5 (highest) were 32.2%, 41.6%, 45.6%, 44.4%, and 46.3%, respectively. The ASIR of CRC for income quintiles 1–5 were 57.5, 54.6, 54.2, 54.6, and 51.1 per 100,000 (standardized to the 2011 Canadian population), respectively. The OncoSim simulation showed that, compared to the status quo scenario, increasing the CRC screening participation rate to 60% would prevent 69,100 (Fig. 2a) and 36,600 CRC cases and deaths respectively (Fig. 2b) across all income quintiles over a 50-year period (2024–2073). Income quintile 1 (Q1) accounted for 32% of the total prevented CRC cases and CRC deaths (Fig. 2a and 2b). Our findings showed that increased CRC screening participation would impact income quintile 1’s CRC incidence and mortality rates the most. Upon increasing the CRC screening rate to 60% between 2024 and 2073, we observed that the majority of the impact would occur in the fifth decade (2064–2073), with 44% of CRC cases prevented and 43% of CRC-related deaths averted during that period (Table 1). Of the 30,175 CRC cases prevented between 2064 and 2073, Q1 would account for the greatest number of cases (9779), while income quintile 5 (Q5) would account for the least number of cases (4293) (Table 1). The number of cases prevented did not follow a gradient corresponding to income quintile, as income quintiles 2, 3, and 4 accounted for 6148, 4751, and 5204 of the prevented cases between 2064 and 2073, respectively (Table 1). The numbers of CRC deaths prevented between 2064 and 2073 across income quintiles 1–5 were estimated as 5104, 3230, 2523, 2743, and 2227, respectively (Table 1).

**Table 1:** Difference in the projected CRC incidence and mortality at a 60% CRC screening participation rate compared to status quo by income quintile in Canada between the periods 2024–2033 (first decade), 2034–2043 (second decade), 2044–2053 (third decade), 2054–2063 (fourth decade), 2064–2073 (fifth decade), and 2024–2073 (entire period)

CRC screening outcomes	Income quintile	First decade (2024–2033)^a^	Second decade (2034–2043)	Third decade (2044–2053)	Fourth decade (2054–2063)	Fifth decade (2064–2073)	Entire period (2024–2073)
CRC cases	All quintiles	140	−3106	−12,108	−23,851	−30,175	−69,100
	1	41	−984	−3848	−7671	−9779	−22,241
	2	22	−639	−2488	−4872	−6148	−14,125
	3	24	−492	−1934	−3783	−4751	−10,936
	4	29	−547	−2120	−4122	−5204	−11,964
	5	24	−444	−1718	−3403	−4293	−9834
CRC deaths	All quintiles	−187	−1802	−6394	−12,397	−15,827	−36,607
	1	−61	−591	−1996	−3973	−5104	−11,725
	2	−38	−371	−1319	−2520	−3230	−7478
	3	−29	−279	−1030	−1969	−2523	−5830
	4	−32	−316	−1113	−2160	−2743	−6364
	5	−27	−245	−936	−1775	−2227	−5210

^a^60% screening participation rate implemented in 2024 onwards

### CRC screening costs, treatment costs, and HAPYs

**Fig. 3:**
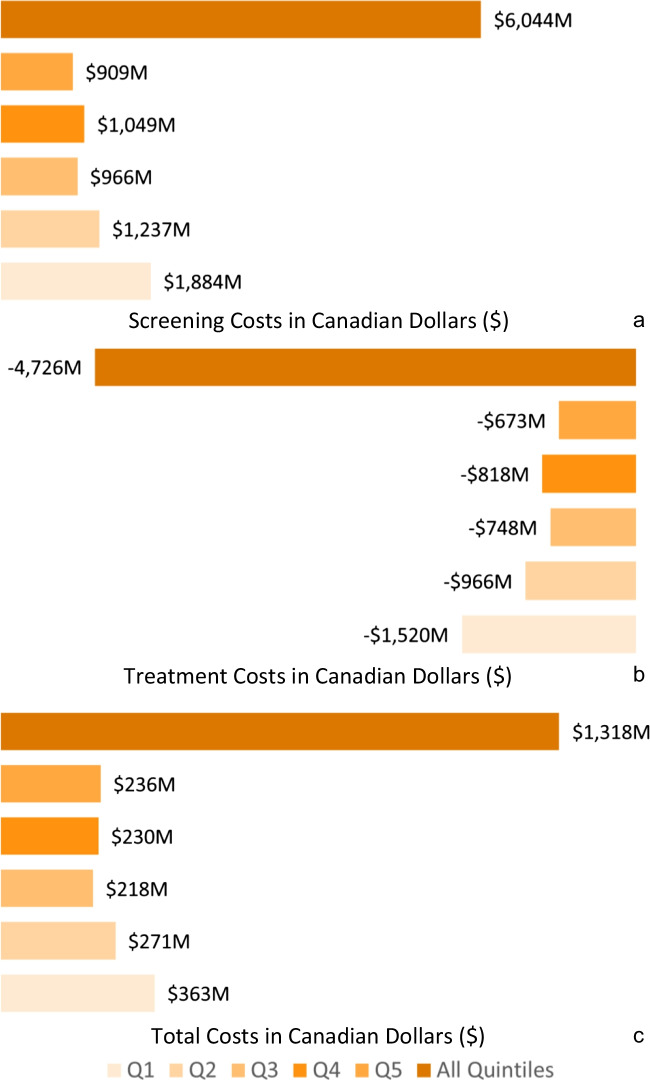
Difference between status quo and projected CRC screening costs, treatment costs, and total costs over a 50-year period (2024–2073) by income quintile in Canada. **a** Difference between status quo and projected CRC screening costs over a 50-year period (2024–2073) by income quintile in Canada. **b** Difference between status quo and projected CRC treatments costs over a 50-year period (2024–2073) by income quintile in Canada. **c** Difference between status quo and projected CRC total costs over a 50-year period (2024–2073) by income quintile in Canada. Q1 = Quintile 1, Q2 = Quintile 2, Q3 = Quintile 3, Q4= Quintile 4, Q5 = Quintile 5, All quintiles = Summation of quintiles 1–5

**Fig. 4:**
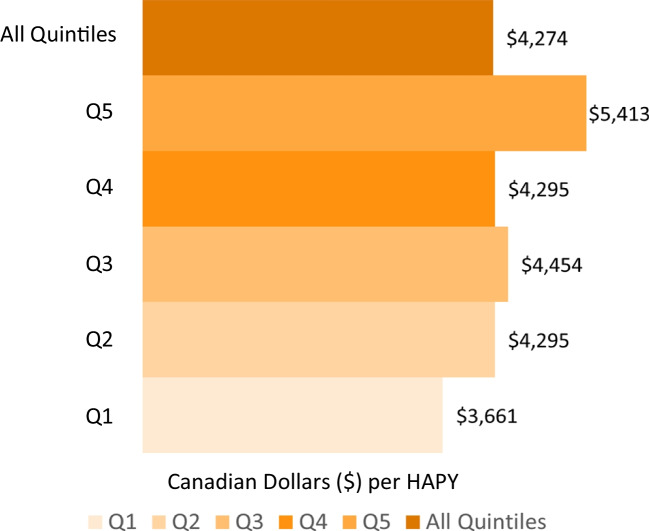
Cost per health-adjusted person year (CAD per HAPY) associated with a 60% CRC screening participation rate over a 50-year period (2024–2073) by income quintile (Q1 = Quintile 1, Q2 = Quintile 2, Q3 = Quintile 3, Q4= Quintile 4, Q5 = Quintile 5, All quintiles = Summation of quintiles 1–5) in Canada

During the period 2024–2073, ramping up the screening participation rate to 60% is projected to lead to an additional $6.0B in screening costs (Fig. 3a). However, the increases in participation would yield $4.7B in treatment-related savings (Fig. 3b), leading to a net additional cost of $1.3B (Fig. 3c). Compared to the other income quintiles, Q1 would be the largest contributor to the additional screening costs ($1.9B) and treatment cost savings ($1.5B) during 2024–2073 (Table 2). Over a 50-year time period (2024–2073), a CRC screening participation rate of 60% is projected to cost $4,274 CAD per health-adjusted person year (HAPY) gained across all income quintiles (Fig. 4). The HAPYs associated with this intervention would cost the most for income quintile 5 ($5,413 CAD per HAPY) and the least for income quintile 1 between 2024 and 2073 ($3,661 CAD per HAPY) (Table 3).

**Table 2:** Difference in the projected CRC screening costs, treatments costs, and total costs at a 60% CRC screening participation rate compared to status quo by income quintile in Canada between the periods 2024–2033 (first decade), 2034–2043 (second decade), 2044–2053 (third decade), 2054–2063 (fourth decade), 2064–2073 (fifth decade), and 2024–2073 (entire period)

CRC screening outcomes	Income quintile	First decade (2024–2033)^a^	Second decade (2034–2043)	Third decade (2044–2053)	Fourth decade (2054–2063)	Fifth decade (2064–2073)	Entire period (2024–2073)
Screening costs (M CAD)	All quintiles	371.1	975.5	1,502.2	1,619.8	1,575.9	6,044.4
	1	115.5	303.9	468.0	505.0	4,91.3	1,883.6
	2	75.8	199.5	307.3	331.4	322.6	1,236.8
	3	59.4	156.0	240.1	258.9	251.8	966.2
	4	64.4	169.2	260.6	281.2	273.4	1,048.8
	5	56.0	146.8	226.2	243.3	236.8	909.1
Treatment costs (M CAD)	All quintiles	−2.3	−230.6	−832.2	−1,604.5	−2,056.7	−4,726.3
	1	−1.3	−72.7	−263.9	−517.0	−665.4	−1,520.1
	2	−0.5	−47.9	−171.4	−326.8	−419.6	−966.2
	3	−0.2	−36.6	−133.1	−253.6	−324.9	−748.4
	4	−0.1	−41.1	−145.2	−276.8	−355.3	−818.4
	5	−0.2	−32.4	−118.6	−230.4	−291.6	−673.2
Total costs (M CAD)	All quintiles	368.8	744.8	670.0	15.3	−480.8	1,318.1
	1	114.2	231.2	204.1	−12.0	−174.1	363.4
	2	75.3	151.7	135.9	4.6	−96.9	270.6
	3	59.2	119.4	107.0	5.3	−73.0	217.9
	4	64.3	128.1	115.4	4.4	−81.9	230.4
	5	55.8	114.4	107.5	12.9	−54.8	235.8

^a^60% screening participation rate implemented in 2024 onwards

**Table 3:** Cost per health-adjusted person year (CAD per HAPY) associated with a 60% CRC screening participation rate by income quintile (All quintiles = Summation of quintiles 1–5, Q1 = Quintile 1, Q2 = Quintile 2, Q3 = Quintile 3, Q4= Quintile 4, Q5 = Quintile 5) in Canada between the periods 2024–2033 (first decade), 2034–2043 (second decade), 2044–2053 (third decade), 2054–2063 (fourth decade), 2064–2073 (fifth decade), and 2024–2073 (entire period)

Cost per HAPY (CAD per HAPY)	First decade (2024–2033)^a^	Second decade (2034–2043)	Third decade (2044–2053)	Fourth decade (2054–2063)	Fifth decade (2064–2073)	Entire period (2024–2073)
All quintiles	280,458	177,514	17,748	146	2,947	4,274
Q1	285,518	161,027	16,939	356	3,318	3,661
Q2	287,463	171,389	17,512	217	2,913	4,295
Q3	276,538	191,643	17,840	322	2,818	4,454
Q4	279,646	176,469	17,391	242	2,889	4,295
Q5	266,901	217,533	20,271	872	2,370	5,413

^a^60% screening participation rate implemented in 2024 onwards

### Sensitivity analysis

We conducted sensitivity analysis with a FIT threshold set to 50 ng/ml and 175 ng/ml. The results of the sensitivity analysis are presented in Supplemental File 1, Tables 8–13. Compared to a FIT threshold of 100 ng/ml, a FIT threshold of 50 ng/ml resulted in 10% more prevented CRC cases and 6% more prevented CRC deaths, while a FIT threshold of 175 ng/ml resulted in 17% fewer prevented CRC cases and 14% fewer prevented CRC deaths.

## Discussion

We observed that increasing the CRC screening participation rate to 60% from the current rate would have a meaningful impact on reducing CRC cases and deaths across all income quintiles over a 50-year span (2024–2073). The most pronounced effect was observed among the lowest income quintile (Q1), which accounted for 32% of the total prevented cases and deaths, highlighting the considerable benefits among lower-income populations. While a CRC screening participation rate of 60% would incur an additional ~$6.0B CAD in screening costs, ~$4.7B of it (78%) would be offset through the savings on treatment costs, resulting in a net additional cost of $1.3B CAD during 2024–2073.

Our results suggest that additional investment into strategies geared towards increasing CRC screening uptake would be most cost-effective for income quintile 1, as the cost per HAPY for this group ($3,661 CAD) was the lowest out of all the income groups. Based on the 2017 CCHS, income quintile 1’s CRC screening participation rate (32%) falls far below the screening rates for income quintiles 2–5 (42–46%). The lower screening participation rate for this group suggests that focused strategies are necessary to increase uptake among this group. While CRC screening is available for all eligible individuals regardless of income, the observed income-related disparities in CRC screening uptake demonstrate that access to such programs is inequitable. To promote equitable access to CRC screening programs and thereby reduce the income-related disparities, additional resources need to be allocated towards targeted interventions that reduce the barriers to screening that are commonly experienced by individuals in the lower income quintiles. Such barriers to screening uptake include a lack of knowledge about the importance of CRC screening, limited time to engage in preventive health measures, and poor health literacy (Decker et al., 2015, 2016; Honein-AbouHaidar et al., 2016); many of which can be further exacerbated by other socioeconomic factors (Pruitt et al., 2009). Currently, the most common initial CRC screening recruitment strategies utilized by CRC screening programs across Canada include health care provider referrals and mailed invitation letters (Canadian Partnership Against Cancer, 2022). These recruitment strategies are further supplemented by reminder notifications, public awareness and social media campaigns, and radio/print advertisements (Canadian Partnership Against Cancer, 2022).

To address the observed disparities in screening uptake, a majority of the CRC screening programs in Canada have implemented a range of targeted strategies aimed at underserved populations. Under many of these programs, educational materials, culturally safe resources, and translated promotional campaigns have all been employed to address the lack of knowledge regarding the risks and benefits of colorectal cancer screening (Canadian Partnership Against Cancer, 2022; Honein-AbouHaidar et al., 2016). While these strategies show promise, additional measures are needed to address the existing gaps in uptake and improve their effectiveness. A previous retrospective cohort study conducted suggested that the use of mailed fecal test kits and health promotion campaigns may have facilitated the elimination of income-related disparities in organized CRC screening programs in Winnipeg (Decker et al., 2016). Several studies conducted in health care centres that primarily serve low-income Hispanics/Latinos in the United States were able to achieve increased CRC screening participation by providing one-on-one CRC educational sessions in clinics, sending invitations to complete an enclosed FIT kit, and following up with routine automated voice and text reminders (Baker et al., 2014; Castañeda et al., 2020). In one such study conducted at a Chicago health care center in which 91% of the population served had incomes below the federal poverty line, a FIT adherence rate of 82% was achieved using a combination of mailed FITs and phone reminders (Baker et al., 2014). Similarly, the direct mailing of an informational postcard followed by an at-home FIT kit to low-income individuals serviced by the San Francisco Health Network resulted in a fit completion rate of 58% compared to the control group’s rate of 37% by the 1-year mark (Somsouk et al., 2020). Although several Canadian CRC screening programs mail testing kits to eligible individuals, in some jurisdictions, the onus is on prospective participants or their primary health care providers to initiate a request for a mailed FIT test. While these at-home fit kits enable individuals to circumvent the potential time barriers associated with attending health care appointments and obtain screenings at their own convenience, this model relies on the assumption of a patient’s proactivity and regular engagement with the health care system.

The primary goal of organized screening is to prevent diseases and improve the outcomes of disease for the entire population through early detection. By screening all eligible individuals, organized programs not only reduce the burden of cancer, but also mitigate the health inequities that are often seen with opportunistic screening. From an equity standpoint, it is crucial to reduce health disparities by investing additional resources in the most underserved population groups. Our analyses suggest that there is also an economic argument for targeting lower income populations. In directing efforts and resources towards lower income groups, health systems could optimize not only population health outcomes, but also cost-effectiveness. While our findings emphasize the substantial benefits of increasing CRC screening participation among lower income individuals, it is important to note that addressing these disparities is a complex undertaking. The strategies employed to increase uptake must be tailored to the specific barriers faced by these populations. Traditional tactics such as invitation letters and provider referrals may not have the same impact within this demographic due to factors such as literacy level, cultural differences, trust in health care providers, and accessibility of health care services. Moreover, while culturally safe materials and interventions have been shown to be beneficial in some contexts, they are not a cure-all (Clifford et al., 2015). Even if materials and messaging are appropriately designed for cultural relevance, there may still be barriers to screening uptake that cannot be addressed by these methods alone. These may include logistical obstacles such as lack of time, difficulties in arranging transportation to appointments, or inflexible work schedules that make it challenging to attend screenings. In the Wequedong Lodge Cancer Screening Program, the supplementation of a culturally sensitive education toolkit with a First Nations Liaison helped to address the negative attitudes towards the health care system that often dissuade members of this group from engaging with cancer screening (Chow et al., 2020). While this individual did not identify as Indigenous, their years of experience serving Indigenous patients provided them with the skills needed to address the cultural barriers that prevent Indigenous peoples from being screened (Chow et al., 2020). Our findings highlight the substantial potential of CRC screening programs to reduce cancer cases and deaths, particularly among lower income populations. These results provide essential insights into how improvements in health outcomes can be maximized through the effective targeting of resources. The challenge lies in identifying and implementing the right mix of interventions to increase screening participation rates effectively among these underserved populations.

### Limitations

The estimates presented here are based on microsimulation modeling data. Although the parameters in the OncoSim model were estimated from the most reliable and representative data sources (Gauvreau et al., 2017), the projections of cancer incidence rate, mortality rate, and management cost to year 2050 rely on the assumption that the past trends of CRC incidence, death, and cost will continue during the projection period. This assumption faces the challenge of the unpredictability of the future. For example, the COVID-19 pandemic caused substantial interruptions and backlogs of the CRC screening programs among most Canadian provinces (Lee et al., 2023); however, OncoSim has not yet incorporated this impact into the CRC model. Another limitation is that the screening costs estimated from these analyses do not include the cost associated with the strategies used to increase the participation rate; therefore, additional costs may be underestimated. While these costs are not included in the models, the considerable gap in the cost per HAPY between the highest and lowest income quintiles suggests that promotional costs to increase participation would have to be considerable to offset the differences.

## Conclusion

Targeting the CRC screening participation rates to 60% will improve health outcomes for individuals across the income spectrum, with the greatest impact observed among the lowest income quintiles in Canada. Overall, this intervention will be the most cost-effective and beneficial for individuals in the lowest income quintile. Although this intervention will incur additional screening costs, its implementation will reduce the treatment costs associated with CRC, as more individuals will be diagnosed at an earlier, more treatable stage.

## Contributions to knowledge

What does this study add to existing knowledge?

- Access barriers at the patient, provider, and health system levels have contributed to disparities in colorectal cancer (CRC) screening uptake in Canada. Among other factors, low household income has been strongly associated with low CRC screening participation.
- This study uses the OncoSim microsimulation platform to project the impact of a screening participation rate of 60% on CRC health and economic outcomes among Canadians in different household income quintiles.
- We found that increased screening will improve health outcomes for all individuals irrespective of household income. This intervention would be most cost-effective for individuals in the lowest income quintile.

What are the key implications for public health interventions, practice, or policy?

- Targeted interventions aimed at increasing CRC screening participation could reduce income-related screening inequities.
- Additional investment in strategies to increase CRC screening uptake could reduce the economic burden of CRC, as the diagnosis of more individuals at an early, more treatable stage will reduce CRC treatment costs.

### Supplementary Information

Below is the link to the electronic supplementary material.

Supplementary file1 (DOCX 329 KB)

## Data Availability

Not applicable.
