# Supplementary material for: The projected health and economic impact of increased colorectal cancer screening participation among Canadians by income quintile
Source: Can J Public Health. 2024 Mar 19;115(3):384–94. doi: 10.17269/s41997-024-00868-8 (PMC11133258; doi:10.17269/s41997-024-00868-8)
Supplement: Supplementary file 1 — Supplementary file1 (DOCX 329 KB) [file 41997_2024_868_MOESM1_ESM.docx]

The Projected Health and Economic Impact of Increased Colorectal Cancer Screening Participation Among Canadians by Income Quintile

*Electronic Supplementary Materials*

The enclosed materials provide further context regarding the inputs and assumptions for the

OncoSim-Colorectal Model.

**Journal Name**

Canadian Journal of Public Health

**Contents**

[Colorectal Cancer Model 4](#_Toc156290402)

[*Natural history of colorectal cancer* 4](#_Toc156290403)

[*Colorectal cancer screening follow-up of abnormal tests* 6](#_Toc156290404)

[*Colorectal cancer survival* 6](#_Toc156290405)

[Screening Regimens 7](#_Toc156290406)

[FIT Sensitivity Analysis 8](#_Toc156290407)

[References 13](#_Toc156290408)

**Tables**

Supplementary Table 1. Distribution of polyp and site by sex, age, and whether the polyp is villous 4

Supplementary Table 2. Sensitivity inputs for distal (descending, sigmoid, rectum) and proximal colon (cecum, ascending, transverse) according to screening tool 5

Supplementary Table 3. Complications of colonoscopy - event probabilities per colonoscopy 5 Supplementary Table 4. Net colorectal cancer survival (OncoSim vs. Canadian Cancer Registry) 6 Supplementary Table 5. Colorectal model inputs 7

Supplementary Table 6. Colorectal cancer screening regimens simulated by risk group from the OncoSim microsimulation model 7

Supplementary Table 7. Positivity rate of FIT by disease status and FIT threshold. Positivity rate is defined as the probability of a positive FIT result among the patients with certain disease status given the specified FIT threshold. 8

Supplementary Table 8. Difference in the projected CRC incidence and mortality at a 60% CRC screening participation rate compared to status quo by income quintile in Canada at a FIT threshold of 50 ng/ml between 2024-2033 (first decade), 2034-2043 (second decade), 2044-2053 (third decade), 2054-2063 (fourth decade), 2064-2073 (fifth decade), and 2024-2073 (entire period). 8

Supplementary Table 9. Difference in the projected CRC screening costs, treatments costs and total costs at a 60% CRC screening participation rate compared to status quo by income quintile in Canada at a FIT threshold of 50 ng/ ml between 2024-2033 (first decade), 2034-2043 (second decade), 2044-2053 (third decade), 2054-2063 (fourth decade), 2064-2073 (fifth decade), and 2024-2073 (entire period). 9

Supplementary Table 10. Cost per Health-adjusted life year (CAD per HALY) associated with a 60% CRC screening participation rate by income quintile (All Quintiles = Summation of Quintiles 1-5, Q1 = Quintile 1, Q2 = Quintile 2, Q3 = Quintile 3, Q4= Quintile 4, Q5 = Quintile 5) in Canada at a FIT threshold of 50 ng/ml between 2024-2033 (first decade), 2034-2043 (second decade), 2044-2053 (third decade), 2054-2063 (fourth decade), 2064-2073 (fifth decade), and 2024-2073 (entire period). 10

Supplementary Table 11. Difference in the projected CRC incidence and mortality at a 60% CRC screening participation rate compared to status quo by income quintile in Canada at a FIT threshold of 175 ng/ml between 2024-2033 (first decade), 2034-2043 (second decade), 2044-2053 (third decade), 2054-2063 (fourth decade), 2064-2073 (fifth decade), and 2024-2073 (entire period). 10

Supplementary Table 12. Difference in the projected CRC screening costs, treatments costs and total costs at a 60% CRC screening participation rate compared to status quo by income quintile in Canada at a FIT threshold of 175 ng/ml between 2024-2033 (first decade), 2034-2043 (second decade), 2044-2053 (third decade), 2054-2063 (fourth decade), 2064-2073 (fifth decade), and 2024-2073 (entire period). 11

Supplementary Table 13. Cost per Health-adjusted life year (CAD per HALY) associated with a 60% CRC screening participation rate by income quintile (All Quintiles = Summation of Quintiles 1-5, Q1 = Quintile 1, Q2 = Quintile 2, Q3 = Quintile 3, Q4= Quintile 4, Q5 = Quintile 5) in Canada at a FIT threshold of 175 ng/ml between 2024-2033 (first decade), 2034-2043 (second decade), 2044-2053 (third decade), 2054-2063 (fourth decade), 2064-2073 (fifth decade), and 2024-2073 (entire period). 12

**Figures**

Supplementary Figure 1. Schematic diagram of the OncoSim-Colorectal model 5

Supplementary Figure 2. OncoSim’s projected overall survival after colon cancer diagnosis (a) colon cancer; (b) rectal cancer 6

# Colorectal Cancer Model

## *Natural history of colorectal cancer*

The model simulates the development of polyps in various sites (cecum, ascending, transverse, descending, sigmoid and rectum) using a distribution (Supplementary Table 1); the probability varies by age and sex. The estimates came from a literature review on adenomatous polyp prevalence, incidence, growth rates, variation by sex, size, site distribution, and histology.^1^

Supplementary Table 1. Distribution of polyp and site by sex, age, and whether the polyp is villous.

| **Sex** | **Age** | **Cecum** | **Ascending** | **Transverse** | **Descending** | **Sigmoid** | **Rectum** |
| --- | --- | --- | --- | --- | --- | --- | --- |
| Non-villous adenoma | | | | | | | |
| **Female** | [min,60[ | 0.03 | 0.32 | 0.22 | 0.13 | 0.19 | 0.10 |
|  | [60,70[ | 0.05 | 0.25 | 0.30 | 0.07 | 0.25 | 0.07 |
|  | [70,80[ | 0.06 | 0.25 | 0.32 | 0.12 | 0.19 | 0.06 |
|  | [80,max] | 0.09 | 0.25 | 0.34 | 0.12 | 0.18 | 0.02 |
| **Male** | [min,60[ | 0.02 | 0.23 | 0.16 | 0.18 | 0.27 | 0.14 |
|  | [60,70[ | 0.04 | 0.19 | 0.23 | 0.10 | 0.34 | 0.10 |
|  | [70,80[ | 0.05 | 0.21 | 0.27 | 0.15 | 0.24 | 0.08 |
|  | [80,max] | 0.09 | 0.25 | 0.34 | 0.12 | 0.18 | 0.02 |
| Villous adenoma* | | | | | | | |
| **Female** | [min,60[ | 0.02 | 0.19 | 0.13 | 0.20 | 0.31 | 0.16 |
|  | [60,70[ | 0.03 | 0.14 | 0.17 | 0.12 | 0.42 | 0.12 |
|  | [70,80[ | 0.03 | 0.13 | 0.17 | 0.21 | 0.34 | 0.11 |
|  | [80,max] | 0.04 | 0.12 | 0.17 | 0.25 | 0.38 | 0.04 |
| **Male** | [min,60[ | 0.02 | 0.19 | 0.13 | 0.20 | 0.31 | 0.16 |
|  | [60,70[ | 0.03 | 0.14 | 0.17 | 0.12 | 0.42 | 0.12 |
|  | [70,80[ | 0.03 | 0.13 | 0.17 | 0.21 | 0.34 | 0.11 |
|  | [80,max] | 0.04 | 0.12 | 0.17 | 0.25 | 0.38 | 0.04 |

*This category includes tubulovillous adenomas because those adenomas are tubulare and villous.


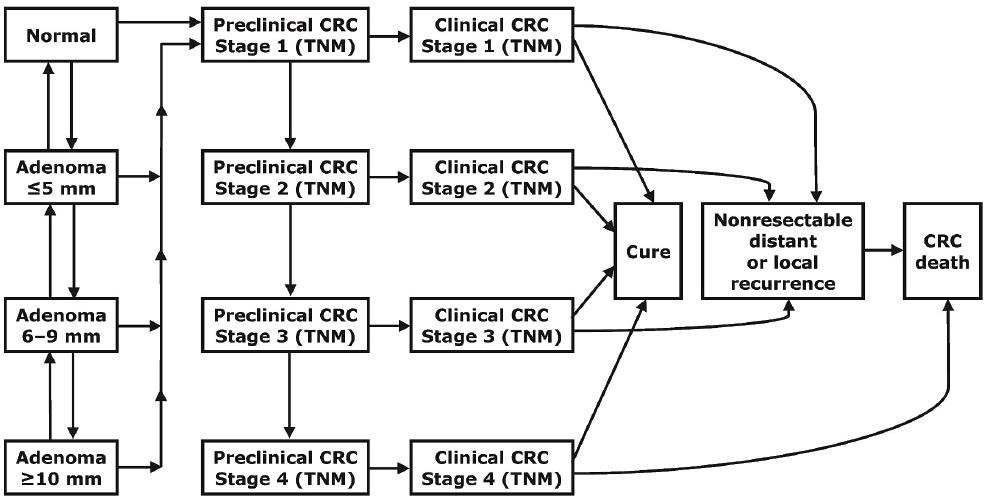


Supplementary Figure 1. Schematic diagram of the OncoSim-Colorectal model^2^

Supplementary Table 2. Sensitivity inputs for distal (descending, sigmoid, rectum) and proximal colon (cecum, ascending, transverse) according to screening tool^1^

|  | **FIT immunochemical** | **Colonoscopy** |
| --- | --- | --- |
| Polyp less or equal to 5mm in size | 0.04 | 0.75 |
| Polyp between 6 and 9mm in size | 0.1 | 0.85 |
| Polyp greater or equal to 10mm in size | 0.3 | 0.95 |
| Cancer | 0.75 | 0.95 |

Supplementary Table 3. Complications of colonoscopy - event probabilities per colonoscopy^3,4,5^

| **Complications of colonoscopy** | **Event rate per colonoscopy** |
| --- | --- |
| Death | 0.0002 |
| Perforation | 0.0017 |
| Haemorrhage | 0.0003 |
| Infection | 0 |
| Cardiopulmonary event | 0 |

## *Colorectal cancer screening follow-up of abnormal tests*

After colonoscopy investigation, subjects are classified into four groups: adenoma-free, low risk, high risk, and cancer. Low risk subjects have fewer than 3 small (<10 mm) nonvillous adenomas and receive another colonoscopy in 5 years; if clear, they then return to screening. High-risk subjects, defined as having 3 or more small adenomas, 1 or more large adenomas (≥10 mm) or an adenoma with a villous or tubulovillous component, receive colonoscopies 3 years after the first follow-up colonoscopy and 5 years after the subsequent colonoscopy. Subjects with cancer receive a colonoscopy the next year and every 3 years thereafter. All adenomas identified at colonoscopy are assumed to be successfully treated.

## *Colorectal cancer survival*

OncoSim’s projected overall survival by stage for colon and rectal cancers are shown in Supplementary Figure 2. As an external validation exercise, we compared OncoSim’s projected net cancer survival with the latest Canadian Cancer Registry data in the Canadian Cancer Statistics report^6^. OncoSim’s projections were similar to the observed estimates (Supplementary Table 4).


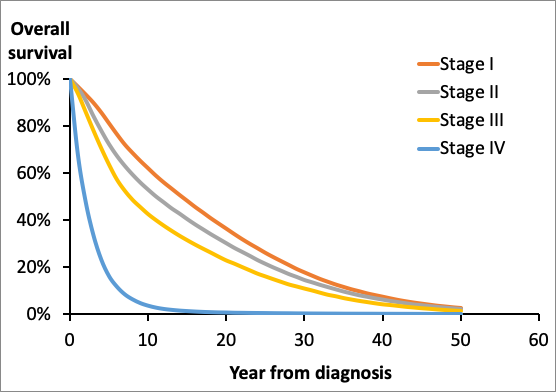


(a) Rectal cancer

**Overall**

**survival**

100%

(b) Colon cancer

80%

Stage I

Stage II Stage III Stage IV

60%

40%

20%

0%

0

10

20

30

40

50

60

**Year from diagnosis**

Supplementary Figure 2. OncoSim’s projected overall survival after colon cancer diagnosis (a) colon cancer; (b) rectal cancer

Data source: Calibrated to match data in the Canadian Cancer Registry

Supplementary Table 4. Net colorectal cancer survival (OncoSim vs. Canadian Cancer Registry)

|  | **OncoSim** | **CCS 2019***  **Mean (95% confidence interval)** |
| --- | --- | --- |
| **5-year net survival** | 66% | 65% (65-66) |
| **10-year net survival** | 59% | 60% (59-61) |

*Data from 2012-2014, excluding Quebec

Supplementary Table 5. Colorectal model inputs

| **Variable** | **Estimate** | **Source** |
| --- | --- | --- |
| Colorectal cancer FIT screening participation rate* | 60% | CCHS ^7^ |
| Adherence rate for follow-up colonoscopy after FIT | 85% | CCHS ^7^ |
| Colonoscopy screening participation rate for individuals with family history of colorectal cancer** | 40% | Assumption |
| **Sensitivity of FIT (threshold: 100ng/mL)** |  |  |
| Small adenoma (<=5 mm) | 0.04 | Coldman et al. (2015) ^1^ |
| Medium adenoma (6-9 mm) | 0.1 | Coldman et al. (2015) ^1^ |
| Large adenoma (>=10 mm) | 0.3 | Coldman et al. (2015) ^1^ |
| Cancer | 0.75 | Coldman et al. (2015) ^1^ |
| Specificity of FIT | 0.96 | Coldman et al. (2015) ^1^ |
| **Sensitivity of colonoscopy** |  |  |
| Small adenoma (<=5 mm) | 0.75 | Coldman et al. (2015) ^1^ |
| Medium adenoma (6-9 mm) | 0.85 | Coldman et al. (2015) ^1^ |
| Large adenoma (>=10 mm) | 0.95 | Coldman et al. (2015) ^1^ |
| Cancer | 0.95 | Coldman et al. (2015) ^1^ |
| Specificity of colonoscopy | 1 | Coldman et al. (2015) ^1^ |

*Simulations with the CRC screening participation rate were carried out at status quo (baseline rate estimated from CCHS 2016) or linearly increasing from the baseline participation rate to 60% between 2020-2029. From 2029 onwards, screening participations rates were held at 60%.

** Assumed 50% of those with family history of colorectal cancer would receive colonoscopy screening, and 80% of them would participate in subsequent screening (every 5 years). The remaining persons with family history would be recruited to participate in the average risk FIT screening program (see participation rate above).

# Screening Regimens

Supplementary Table 6. Colorectal cancer screening regimens simulated by risk group from the OncoSim microsimulation model

|  | **Colorectal** | |
| --- | --- | --- |
| **Risk group** | **Average risk** | **Elevated**** |
| **Test** | Fecal immunochemical test (FIT) | Colonoscopy |
| **Interval** | 2 years | 5 years |
| **Screening ages** | 50-74 years | 40-74 years |

Abbreviations: FIT, fecal immunochemical test

** People with one or more first degree family history of colorectal cancer; we assumed 50% of elevated risk individuals are screened with this regimen, and the remaining 50% are invited to screen with the average risk regimen.

# FIT Sensitivity Analysis

Supplementary Table 7. Positivity rate of FIT by disease status and FIT threshold. Positivity rate is defined as the probability of a positive FIT result among the patients with certain disease status given the specified FIT threshold.

| **Polyp/Cancer state** | **FIT 50 ng/ml** | **FIT 100 ng/ml** | **FIT 175 ng/ml** |
| --- | --- | --- | --- |
| No polyp or cancer | 0.07 | 0.04 | 0.02 |
| Polyp <= 5mm | 0.14 | 0.11 | 0.07 |
| Polyp 6-9 mm | 0.21 | 0.17 | 0.12 |
| Polyp <=10mm | 0.32 | 0.27 | 0.20 |
| Cancer | 0.85 | 0.78 | 0.64 |

Supplementary Table 8. Difference in the projected CRC incidence and mortality at a 60% CRC screening participation rate compared to status quo by income quintile in Canada at a FIT threshold of 50 ng/ml between 2024-2033 (first decade), 2034-2043 (second decade), 2044-2053 (third decade), 2054-2063 (fourth decade), 2064-2073 (fifth decade), and 2024-2073 (entire period).

| CRC Screening Outcomes | Income Quintile | First decade (2024-2033)^a^ | Second decade (2034-2043) | Third decade (2044-2053) | Fourth decade (2054-2063) | Fifth decade (2064-2073) | Entire Period (2024-2073) |
| --- | --- | --- | --- | --- | --- | --- | --- |
| CRC cases | All Quintiles | 108 | -3,730 | -13,372 | -25,991 | -32,814 | -75,799 |
|  | 1 | 27 | -1,170 | -4,279 | -8,365 | -10,634 | -24,421 |
|  | 2 | 21 | -764 | -2,743 | -5,309 | -6,682 | -15,477 |
|  | 3 | 18 | -598 | -2,135 | -4,127 | -5,175 | -12,017 |
|  | 4 | 23 | -656 | -2,334 | -4,494 | -5,659 | -13,120 |
|  | 5 | 19 | -542 | -1,881 | -3,696 | -4,664 | -10,764 |
| CRC deaths | All Quintiles | -198 | -1,948 | -6,766 | -13,101 | -16,663 | -38,676 |
|  | 1 | -64 | -624 | -2,117 | -4,213 | -5,390 | -12,408 |
|  | 2 | -40 | -401 | -1,394 | -2,664 | -3,412 | -7,911 |
|  | 3 | -30 | -308 | -1,092 | -2,085 | -2,638 | -6,153 |
|  | 4 | -34 | -347 | -1,179 | -2,277 | -2,886 | -6,723 |
|  | 5 | -30 | -268 | -984 | -1,862 | -2,337 | -5,481 |

^a^60% Screening Participation rate implemented in 2024 onwards.

Supplementary Table 9. Difference in the projected CRC screening costs, treatments costs and total costs at a 60% CRC screening participation rate compared to status quo by income quintile in Canada at a FIT threshold of 50 ng/ ml between 2024-2033 (first decade), 2034-2043 (second decade), 2044-2053 (third decade), 2054-2063 (fourth decade), 2064-2073 (fifth decade), and 2024-2073 (entire period).

| CRC Screening Outcomes | Income Quintile | First decade (2024-2033)^a^ | Second decade (20234-2043) | Third decade (2044-2053) | Fourth decade (2054-2063) | Fifth decade (2064-2073) | Entire Period (2024-2073) |
| --- | --- | --- | --- | --- | --- | --- | --- |
| Screening costs  (M CAD) | All Quintiles | 418.6 | 1076.2 | 1643.6 | 1769.5 | 1720.8 | 6628.8 |
|  | 1 | 130.3 | 335.3 | 512.1 | 551.6 | 536.3 | 2065.5 |
|  | 2 | 85.6 | 220.2 | 336.3 | 362.2 | 352.3 | 1356.5 |
|  | 3 | 67.0 | 172.1 | 262.6 | 282.9 | 275.1 | 1059.7 |
|  | 4 | 72.7 | 186.7 | 285.1 | 307.2 | 298.5 | 1150.2 |
|  | 5 | 63.1 | 162.0 | 247.5 | 265.7 | 258.7 | 996.9 |
| Treatment costs (M CAD) | All Quintiles | -4.2 | -263.9 | -906.4 | -1727.6 | -2217.8 | -5119.9 |
|  | 1 | -1.8 | -82.5 | -288.6 | -557.4 | -718.3 | -1648.6 |
|  | 2 | -0.9 | -54.5 | -186.5 | -352.2 | -453.4 | -1047.5 |
|  | 3 | -0.5 | -42.2 | -145.0 | -273.3 | -350.2 | -811.3 |
|  | 4 | -0.4 | -47.0 | -157.7 | -297.8 | -382.7 | -885.6 |
|  | 5 | -0.6 | -37.7 | -128.6 | -246.8 | -313.3 | -726.9 |
| Total costs  (M CAD) | All Quintiles | 414.4 | 812.3 | 737.2 | 41.9 | -497.0 | 1508.9 |
|  | 1 | 128.5 | 252.8 | 223.4 | -5.9 | -182.0 | 416.9 |
|  | 2 | 84.6 | 165.6 | 149.8 | 10.0 | -101.0 | 309.0 |
|  | 3 | 66.5 | 129.8 | 117.7 | 9.6 | -75.2 | 248.4 |
|  | 4 | 72.2 | 139.7 | 127.4 | 9.4 | -84.2 | 264.5 |
|  | 5 | 62.6 | 124.3 | 119.0 | 18.8 | -54.6 | 270.1 |

^a^60% Screening Participation rate implemented in 2024 onwards.

Supplementary Table 10. Cost per Health-adjusted person year (CAD per HAPY) associated with a 60% CRC screening participation rate by income quintile (All Quintiles = Summation of Quintiles 1-5, Q1 = Quintile 1, Q2 = Quintile 2, Q3 = Quintile 3, Q4= Quintile 4, Q5 = Quintile 5) in Canada at a FIT threshold of 50 ng/ml between 2024-2033 (first decade), 2034-2043 (second decade), 2044-2053 (third decade), 2054-2063 (fourth decade), 2064-2073 (fifth decade), and 2024-2073 (entire period).

| Cost Per HAPY (CAD per HAPY) | First decade (2024-2033)^a^ | Second decade (2034-2043) | Third decade (2044-2053) | Fourth decade (2054-2063) | Fifth decade (2064-2073) | Entire Period (2024-2073) |
| --- | --- | --- | --- | --- | --- | --- |
| All Quintiles | 215,834 | 226,776 | 18,832 | 380 | 2,883 | 4,662 |
| Q1 | 219,672 | 207,924 | 17,897 | 165 | 3,271 | 3,994 |
| Q2 | 217,548 | 217,376 | 18,630 | 442 | 2,873 | 4,674 |
| Q3 | 212,400 | 238,624 | 18,806 | 547 | 2,749 | 4,834 |
| Q4 | 216,273 | 221,757 | 18,541 | 491 | 2,816 | 4,706 |
| Q5 | 209,199 | 289,109 | 21,645 | 1,209 | 2,243 | 5,928 |

^a^60% Screening Participation rate implemented in 2024 onwards.

Supplementary Table 11. Difference in the projected CRC incidence and mortality at a 60% CRC screening participation rate compared to status quo by income quintile in Canada at a FIT threshold of 175 ng/ml between 2024-2033 (first decade), 2034-2043 (second decade), 2044-2053 (third decade), 2054-2063 (fourth decade), 2064-2073 (fifth decade), and 2024-2073 (entire period).

| CRC Screening Outcomes | Income Quintile | First decade (2024-2033)^a^ | Second decade (2034-2043) | Third decade (2044-2053) | Fourth decade (2054-2063) | Fifth decade (2064-2073) | Entire Period (2024-2073) |
| --- | --- | --- | --- | --- | --- | --- | --- |
| CRC cases | All Quintiles | 171 | -2,266 | -9,806 | -19,997 | -25,343 | -57,241 |
|  | 1 | 54 | -719 | -3,130 | -6,452 | -8,209 | -18,456 |
|  | 2 | 32 | -467 | -2,016 | -4,083 | -5,156 | -11,690 |
|  | 3 | 28 | -359 | -1,552 | -3,167 | -4,000 | -9,050 |
|  | 4 | 31 | -395 | -1,715 | -3,458 | -4,364 | -9,901 |
|  | 5 | 26 | -326 | -1,393 | -2,837 | -3,614 | -8,144 |
| CRC deaths | All Quintiles | -152 | -1,525 | -5,638 | -10,883 | -13,836 | -32,034 |
|  | 1 | -51 | -498 | -1,763 | -3,509 | -4,465 | -10,286 |
|  | 2 | -30 | -316 | -1,160 | -2,204 | -2,819 | -6,529 |
|  | 3 | -23 | -238 | -906 | -1,731 | -2,205 | -5,103 |
|  | 4 | -27 | -267 | -984 | -1,889 | -2,396 | -5,563 |
|  | 5 | -21 | -206 | -825 | -1,550 | -1,951 | -4,553 |

^a^60% Screening Participation rate implemented in 2024 onwards.

Supplementary Table 12. Difference in the projected CRC screening costs, treatments costs and total costs at a 60% CRC screening participation rate compared to status quo by income quintile in Canada at a FIT threshold of 175 ng/ml between 2024-2033 (first decade), 2034-2043 (second decade), 2044-2053 (third decade), 2054-2063 (fourth decade), 2064-2073 (fifth decade), and 2024-2073 (entire period).

| CRC Screening Outcomes | Income Quintile | First decade (2024-2033)^a^ | Second decade (2034-2043) | Third decade (2044-2053) | Fourth decade (2054-2063) | Fifth decade (2064-2073) | Entire Period (2024-2073) |
| --- | --- | --- | --- | --- | --- | --- | --- |
| Screening costs  (M CAD) | All Quintiles | 320.5 | 859.3 | 1336.0 | 1441.8 | 1405.5 | 5363.1 |
|  | 1 | 99.6 | 267.6 | 416.0 | 449.3 | 437.9 | 1670.4 |
|  | 2 | 65.5 | 175.8 | 273.3 | 295.0 | 287.6 | 1097.2 |
|  | 3 | 51.3 | 137.4 | 213.7 | 230.5 | 224.7 | 857.7 |
|  | 4 | 55.6 | 149.1 | 231.8 | 250.2 | 243.9 | 930.6 |
|  | 5 | 48.4 | 129.5 | 201.2 | 216.8 | 211.3 | 807.2 |
| Treatment costs (M CAD) | All Quintiles | 0.8 | -178.8 | -693.7 | -1363.7 | -1742.7 | -3978.2 |
|  | 1 | -0.3 | -56.2 | -221.2 | -442.1 | -563.8 | -1283.7 |
|  | 2 | 0.3 | -37.1 | -142.6 | -277.1 | -355.3 | -811.9 |
|  | 3 | 0.2 | -28.5 | -110.1 | -215.0 | -275.4 | -628.7 |
|  | 4 | 0.4 | -31.8 | -120.7 | -234.7 | -300.4 | -687.1 |
|  | 5 | 0.2 | -25.3 | -99.1 | -194.8 | -247.8 | -566.8 |
| Total costs  (M CAD) | All Quintiles | 321.2 | 680.5 | 642.2 | 78.1 | -337.2 | 1384.9 |
|  | 1 | 99.3 | 211.4 | 194.7 | 7.2 | -125.9 | 386.7 |
|  | 2 | 65.8 | 138.7 | 130.7 | 17.9 | -67.7 | 285.3 |
|  | 3 | 51.6 | 108.9 | 103.6 | 15.5 | -50.6 | 228.9 |
|  | 4 | 56.0 | 117.3 | 111.1 | 15.5 | -56.5 | 243.5 |
|  | 5 | 48.6 | 104.2 | 102.1 | 22.0 | -36.4 | 240.5 |

^a^60% Screening Participation rate implemented in 2024 onwards.

Supplementary Table 13. Cost per Health-adjusted person year (CAD per HAPY) associated with a 60% CRC screening participation rate by income quintile (All Quintiles = Summation of Quintiles 1-5, Q1 = Quintile 1, Q2 = Quintile 2, Q3 = Quintile 3, Q4= Quintile 4, Q5 = Quintile 5) in Canada at a FIT threshold of 175 ng/ml between 2024-2033 (first decade), 2034-2043 (second decade), 2044-2053 (third decade), 2054-2063 (fourth decade), 2064-2073 (fifth decade), and 2024-2073 (entire period).

| Cost Per HAPY (CAD per HAPY) | First decade (2024-2033)^a^ | Second decade (2034-2043) | Third decade (2044-2053) | Fourth decade (2054-2063) | Fifth decade (2064-2073) | Entire Period (2024-2073) |
| --- | --- | --- | --- | --- | --- | --- |
| All Quintiles | 435,295 | 165,606 | 19,228 | 852 | 2,378 | 5,127 |
| Q1 | 453,599 | 152,840 | 18,289 | 242 | 2,754 | 4,439 |
| Q2 | 450,379 | 160,534 | 19,068 | 959 | 2,350 | 5,186 |
| Q3 | 419,107 | 174,528 | 19,490 | 1,071 | 2,246 | 5,341 |
| Q4 | 430,711 | 166,674 | 18,938 | 977 | 2,299 | 5,189 |
| Q5 | 405,099 | 195,039 | 21,644 | 1,695 | 1,810 | 6,291 |

^a^60% Screening Participation rate implemented in 2024 onwards.

# References

1. Coldman AJ, Phillips N, Brisson J, et al. Evaluating colorectal cancer screening options for Canada using the Cancer Risk Management Model. Curr Oncol. 2015 Apr; 22(2):e41- 50.
2. Coldman A, Flanagan W, Nadeau C, Wolfson M, Fitzgerald N, Memon S, et al. Projected effect of fecal immunochemical test threshold for colorectal cancer screening on outcomes and costs for Canada using the OncoSim microsimulation model. J Cancer Policy. 2017 Sep;13:38–46.
3. Habr-Gama A, Waye JD. Complications and hazards of gastrointestinal endoscopy. World J Surg. 1989;13(2):193-201. doi:10.1007/BF01658399
4. Bercy G, et al.: Complications of colonoscopy and polypectomy. Gastroent. 1974. 67: 548.
5. Macrae FA, Tan KG, Williams CB: Towards safer colonoscopy: a report on the complications of 5000 diagnostic or therapeutic colonoscopies. Gut 1983; 24:376-383.
6. Canadian Cancer Statistics Advisory Committee. Canadian Cancer Statistics 2019. Toronto, ON: Canadian Cancer Society; 2019. Available at: cancer.ca/Canadian-Cancer- Statistics-2019-EN (accessed July 1, 2020).
7. Canadian Community Health Survey (CCHS) 2018, Statistics Canada, available from: https://www23.statcan.gc.ca/imdb/p3Instr.pl?Function=assembleInstr&a=1&&lang=en& Item_Id=839130
